# In vivo study of pro-inflammatory cytokine changes in serum and synovial fluid during treatment with celecoxib and etoricoxib and correlation with VAS pain change and synovial membrane penetration index in patients with inflammatory arthritis

**DOI:** 10.31138/mjr.28.1.33

**Published:** 2017-03-28

**Authors:** Athina Theodoridou, Helen Gika, Eudoxia Diza, Alexandros Garyfallos, Loucas Settas

**Affiliations:** 1 4^th^ Department of Internal Medicine Hippokrateion University Hospital, Aristotle University of Thessaloniki Medical School, Thessaloniki, Greece; 2 Rheumatology Division, 1^st^ Department of Internal Medicine, Aristotle University of Thessaloniki Medical School, Thessaloniki, Greece; 3 Forensic Medicine and Toxicology, Aristotle University of Thessaloniki Medical School, Thessaloniki, Greece; 4 B’ Microbiology Dep, AHEPA University Hospital, Aristotle University of Thessaloniki Medical School, Thessaloniki, Greece

**Keywords:** inflammatory arthritis, cytokines, synovial fluid, etoricoxib, celecoxib

## Abstract

**Objectives:**

To determine the impact of celecoxib and etoricoxib therapy on serum and synovial fluid levels of IL-1β, IL-6, TNF-α, sTNFR1, sTNFR2 and IL-1Ra in patients with inflammatory arthritis. To determine the correlation between cytokine changes and synovial membrane penetration index of the study drugs, and pain VAS change.

**Methods:**

Fifty-one patients with inflammatory synovial fluid accumulation in a knee joint (33 women), randomized on 3 groups of 17 each: 100 mg b.i.d. celecoxib treated group, 90 mg o.d. etoricoxib treated group, and the control group with no NSAID treatment. Cytokines serum and synovial fluid levels as well as membrane penetration index were assessed prior and after treatment.

**Results:**

Celecoxib led to decrease of both synovial fluid and serum levels of IL-6 (p=0.017 and p=0.003, respectively). In the etoricoxib treated group synovial fluid IL-6 concentration was significantly decreased after treatment (p=0.019). Correlating the study drugs penetration index with the change of cytokines and their receptors levels, positive correlation was found with the reduction of synovial fluid IL-1β for the celecoxib (p=0.032) and with the increase of synovial fluid sTNFR1 for the etoricoxib group (p=0.028). Pain VAS reduction was positively correlated with decrease of synovial fluid IL-1β (p=0.041) and IL-6 levels (p<0.005) and negative with synovial fluid sTNFR1 changes (p=0.045) in celecoxib group, and negative with serum TNF-α decrease (p=0.044) in the etoricoxib group.

**Conclusion:**

Our results suggest that celecoxib and etoricoxib inhibit the inflammatory cytokines, mostly in synovial fluid but also in serum, causing through this mechanism, decrease of inflammation, irrespective to COX-2 inhibition.

## INTRODUCTION

Since 1897, when aspirin was introduced as the first non-steroidal anti-inflammatory drug,^1^ non-steroidal anti-inflammatory drugs (NSAIDs) are widely used for the control of pain and inflammation. Coxibs are the new-generation NSAIDs, and are selective inhibitors cycloxygenase-2 (COX-2). Their mode of action is not clear yet, as several studies demonstrated anti-inflammatory and antiproliferative effects, independent of cyclooxygenase activity and prostaglandin synthesis inhibition. Their differences in cyclooxygenase-independent mechanisms may have consequences for the specific use of coxibs in individual patients, because these mechanisms may affect either their efficacy or their toxicity. A small number of studies about the influence of celecoxib, etoricoxib and rofecoxib on cytokines and transcription factors have been published, and most of them are either in animals or in cell lines,^2,3^ and few in vivo in humans.^4–6^ Moreover, one of the research goals for the development of new NSAIDs is their effects on signal transduction and their inhibitory action on the inflammatory cytokines.^7,8^ Possibly there is an endogenous weakness of some NSAIDs, which, according to particular data, may have a tendency to promote, in addition to inhibit inflammation.^9^ This pro-inflammatory effect is obvious from the results of both in vivo and in vitro studies in animals and humans, where some NSAIDs caused increase of tumor necrosis factor-alpha (TNF-α). TNF-α mediates in the inflammatory action in many cell populations and induces multiple biochemical pro-inflammatory processes. Additionally, TNF-α induces COX-2, which has a half-life longer than that of TNF-α.^10^ TNF-α is also implicated in the pathogenesis of NSAIDs adverse reactions on the intestine; for instance, indomethacin induces intestine lesions that look inflammatory under a microscope, and the amount of mucosal erosions parallel the local TNF-α amount. Moreover, with use of NSAIDs, the frequent subclinical increase of the small intestine’s mucosa penetration is associated with the increase of TNF-α.^11,12^

The aim of this study was to determine the impact of celecoxib and etoricoxib on the concentrations of IL-1β, IL-6, TNF-α, sTNFR1, sTNFR2 and IL-1Ra in serum and synovial fluid (SF) in individuals with inflammatory knee arthritis as well as the correlation between cytokines concentration changes, with study drugs concentrations, coxibs’ synovial membrane (SM) penetration index and pain VAS changes.

## MATERIALS AND METHODS

### Study population

Ninety-eight patients were screened. The Inclusion Criteria were 1) age 18–80 years, 2) SF accumulation in a knee joint, in a patient with inflammatory arthritis like rheumatoid arthritis, psoriatic arthritis, seronegative spondyloarthritis, undifferentiated arthritis, or inflammatory osteoarthritis, 3) SF, white cell count (WBC) >2000/mm^3^. The exclusion criteria were 1) septic arthritis either during recruitment, or during the study, 2) treatment with biologic agents and treatment modifications of disease modifying antirheumatic drugs (DMARDs) in the last 2 months, 3) treatment with NSAIDs in the last 2 weeks before study entry, 4) any kind of inflammation in the body with infectious etiology or not, 5) intraarticular injections in the knee from which synovial fluid was drown from, in the last 2 months, 6) history of hypersensitivity to aspirin, coxibs, or other NSAID, 7) Surgical intervention of the knee in particular the last 6 months before study entry, 8) knee trauma within the last 6 months, and 9) SF values of WBC ≤2000mm or ≥50.000mm.

From the 98 patients screened, 51 (**Table 1**) fulfilled inclusion and exclusion criteria, and were randomized in three groups age and gender matched: In group A, patients received celecoxib 100 mg b.i.d, in group B patients received, etoricoxib 90 mg o.d and in group C, patients received no NSAID during study. No change of concomitant medications was allowed to patients of all groups and use of paracetamol as a rescue drug was allowed only to group C patients and was discontinued 48 hours before the second visit. During the first visit, detailed medical history was taken and physical examination of the patients was performed. Also, a visual analogue scale (VAS) for pain was completed by the patients. Then knee joint aspiration was performed and 10 ml blood were collected from every patient. Immediately after aspiration, SF was cultured, a WBC count and differential was done, and at least 5 ml of SF were centrifuged in heparinized tube and were stored at −70. Blood serum was also stored at −70. After that, every patient was randomized in one of the study groups and started the appropriate treatment. Control group received no NSAID or drug other than allowed by protocol, whereas paracetamol was allowed as a rescue drug, only to be discontinued 48 hours before second visit. Patients came back for the second visit seven days later so that steady state conditions could be achieved. We repeated the same procedures as during the first visit, 3 hours after the morning dose of celecoxib and 1 hour after the daily dose of etoricoxib, in order to have C^*max*^ of the study drugs according to their known pharmacokinetics. The serum and joint fluid collection were done in steady state conditions in all cases.

**Table 1.** Demographic Data

**Parameter**		**Celecoxib group**	**Etoricoxib group**	**Control group**	**p**
**Sex** n (%)	male	5 (29.4)	9 (52.9)	4 (23.5)	0.16
	female	12 (70.6)	8 (47.0)	13 (76.4)	
**Age** Mean value (SD)		56.2 (17.4)	57.2 (19.2)	67.3 (9.9)	0.19
**BMI** Mean value (SD)		25.9 (6.5)	24.7 (3.1)	28.5 (2.7)	**0.01**

n: number, BMI: body mass index, SD: standard deviation

## METHODS

(i) Determinations of Celecoxib and Etoricoxib Concentrations in SF and Serum were performed as analytically described in our previous paper. Briefly High Performance Liquid Chromatography was coupled to Inductively Coupled Plasma Mass Spectrometry for the measurement of sulphur containing compounds, such as both drug compounds. Ultra Performance Liquid Chromatography was coupled to Quadrupole Time of Flight Mass Spectrometry to quantitate and detect drug metabolites.^13^

(ii) Penetration index of the study drugs was determined in every patient according to the following form: penetration index=SF concentration/serum concentration ×100.

(iii) Determination of cytokines and their soluble receptors in serum and SF: We determined levels of IL-1β, IL-6, TNF-α and soluble cytokines sTNFR1 sTNFR2 and IL-1Ra using Sandwich ELISA method (R & D Systems-Minneapolis USA).

*IL-1β determination:* According to the manufacturer, the intra assay CV is 2.8–8.5 % day, the inter assay CV is 4.1–8.4 %, the sensitivity of this method less than 1pg/ml, and the specificity is very high. In every sample of either serum or synovial fluid where IL-1β could not be detected, we used high sensitivity kits. According to the manufacturer in these high sensitivity kits, the intra assay CV is 4.3–10.2 %, the inter assay CV is 7.3–10.4 %, the sensitivity is from 0.023–0.140 pg/ml, and the specificity is very high.

*IL-6 determination:* According to the manufacturer, the intra assay CV is 1.6–4.2 %, the inter assay CV 3.3–6.4 %, the sensitivity of this method is less than 0.7pg/ml, and the specificity is very high. In every sample of either serum or synovial fluid where IL-6 could not be detected, we used high sensitivity kits. According to the manufacturer, in these high sensitivity kits the intra assay CV is 6.9–7.8 %, the inter assay CV 6.5–9.6 %, the sensitivity is from 0.447–9.96pg/ml, and the specificity is very high.

*TNF-α determination:* According to the manufacturer, the intra assay CV is 4.2–.2 %, the inter assay CV 4.6–7.4 %, the sensitivity of these reagents is 0.5–5.5pg/ml, and the specificity is very high. In every sample of either serum or synovial fluid where TNF-α could not be detected, we used high sensitivity kits. According to the manufacturer, in these high sensitivity kits the intra assay CV is 3.1–8.5 %, the inter assay CV 7.3–10.6%, the sensitivity is from 0.038–0.191pg/ml, and the specificity is very high.

*IL-1Ra determination:* According to the manufacturer, the intra assay CV is 3.1–6.2%, the inter assay CV 4.4–6.7 %, the sensitivity of these reagents is 14pg/ml, and the specificity is very high.

*sTNFRI (55 kDa) determination:* According to the manufacturer, the intra assay CV is 3.6–5.0 %, the inter assay CV 3.7–8.8 %, the sensitivity of these reagents is 0.43–1.2pg/ml, and the specificity is very high.

*sTNFRI (55 kDa) determination:* According to the manufacturer, the intra assay CV is 2.6–4.8 %, the inter assay CV 3.5–5.1 %, the sensitivity of these reagents is 0.2–2.3pg/ml, and the specificity is very high.

(iv) Statistical Analysis. For data presentation, descriptive tests like mean value, standard deviation, median value and interquartile range were used. Median value and interquartile range were selected for cytokines, as cytokines are characterized for the display of extreme values which results on the restricted potentiality of mean value and standard deviation to depict variables of data.

For correlation analyses either between study groups, or between measurements of the same variable, absolute values were used for the clinical variables and percentage of changes were used for cytokines. The reason for this discrimination was the extreme values of cytokine measurements.

When comparison was among the three study groups Kruskal-Wallis [436^α^] was used, and when comparison was between first and second visit measurement of the same variable of a particular study group Wilcoxon test [436^β^] was used for statistic control. The use of non-parametric methods was selected due the relatively small statistical sample. For correlations analysis, Pearson’s correlation coefficient was used. Analysis of data was processed by SAS 9.1.3 software (SAS Institute Inc., Cary, NC, USA). Level of significance was determined at 5%.

## RESULTS

(i) Cytokine levels in serum and SF: In the celecoxib treated group, we observed a statistical significant: increase in SF IL-1Ra (p<0.001), decrease in SF IL-6 (p=0.017) and decrease in serum IL-6 (p=0.003). In the etoricoxib treated group we observed statistical significant: decrease of serum IL-1β (p=0.045), decrease of SF IL-6 (p=0.019) and increase of SF sTNFR2 (p=0.017). In the control group, we observed a significant increase of IL-1β (p=0.001) and TNF-α (p=0.01) levels in SF (**Table 2**).

**Table 2:** Median values of cytokines and cytokine receptors in the 1^st^ and 2^nd^ visit before and after the treatment with celecoxib and etoricoxib in the respective group and in the control group. Values are in pg/ml

	**Celecoxib group**			**Etoricoxib group**			**Control group**		
	**1^st^ visit Median (IQR)**	**2^nd^ visit Median (IQR)**	**p**	**1^st^ visit Median (IQR)**	**2^nd^ visit Median (IQR)**	**p**	**1^st^ visit Median (IQR)**	**2^nd^ visit Median (IQR)**	**p**
**IL1**β **S**	0.24 (0.56)	0.15 (0.12)	NS	0.45 (0.51)	0.23 (0.18)	**0.045**	0.19 (0.19)	0.21 (0.31)	NS
**IL1β JF**	0.65 (0.92)	0.20 (0.62)	NS	2.30 (5.77)	1.60 (5.85)	NS	0.40 (0.43)	0.54 (0.88)	**0.001**
**IL1Ra S**	240.0 (180.0)	250.0 (110.0)	NS	250.0 (195.0)	230.0 (295.0)	NS	460.0 (740.0)	710.0 (940.0)	NS
**IL1Ra JF**	340.0 (2210.0)	460.0 (5000.0)	**<0.001**	460.0 (8210.0)	1100.0 (8600.0)	NS	450.0 (1475.0)	400.0 (1095.0)	NS
**IL6 S**	1.30 (2.34)	1.20 (1.67)	**0.003**	1.70 (9.38)	1.35 (9.55)	NS	1.65 (1.10)	1.60 (1.80)	NS
**IL6 JF**	500.0 (5010.0)	420.0 (2065.0)	**0.017**	1450.0 (11360.0)	880.0 (3140.0)	**0.019**	300.0 (1300.0)	300.0 (1340.0)	NS
**TNF-α S**	1.20 (0.60)	1.10 (0.40)	NS	1.70 (1.00)	1.10 (1.40)	NS	1.30 (0.40)	1.30 (0.35)	NS
**TNF-α JF**	1.50 (2.89)	1.00 (3.10)	NS	2.90 (5.15)	1.40 (3.70)	NS	1.10 (1.25)	1.70 (2.80)	**0.01**
**sTNFR1 S**	1650.0 (500.0)	1450.0 (450.0)	NS	2000.0 (1200.0)	1650.0 (1350.0)	NS	1550.0 (1300.0)	1450.0 (750.0)	NS
**sTNFR1 JF**	5000.0 (4500.0)	7200.0 (4000.0)	NS	7200.0 (5600.0)	8400.0 (4600.0)	NS	7200.0 (7300.0)	5600.0 (6300.0)	NS
**sTNFR2 S**	3000.0 (600.0)	2800.0 (500.0)	NS	3300.0 (1000.0)	2800.0 (600.0)	NS	3400.0 (2800.0)	3300.0 (2950.0)	NS
**sTNFR2 JF**	3500.0 (4400.0)	4250.0 (3400.0)	NS	4200.0 (5800.0)	5750.0 (9000.0)	**0.017**	4800.0 (5450.0)	3600.0 (4200.0)	NS

NS: non significant, IQR: interquartile range, s: soluble, S: serum, JF: joint fluid

(ii) Correlations between changes of cytokines, receptors and pain VAS, drug concentrations and penetration index. **Table 3** depicts correlations between serum and SF drug concentrations, with changes of VAS and percentage of changes of cytokines and receptors from 1^st^ to 2^nd^ visit. Regarding celecoxib serum concentration correlations, we noticed a statistically important positive correlation with sTNFR2 in SF (p=0.028, correlation coefficient=0.282), and a marginal correlation with serum IL-1β (p=0.077, correlation coefficient=0.324) and serum sTNFR1 (p=0.051, correlation coefficient=0.182). Regarding celecoxib SF concentration correlations, we noticed a statistically important positive correlation with SF IL-1β (p=0.032, correlation coefficient=0.238), and a marginal correlation with SF IL-1Ra (p=0.071, correlation coefficient=0.451). Etoricoxib serum concentrations were not related with cytokines but etoricoxib SF concentration were inversely correlated with SF IL-1β (p=0.005, correlation coefficient=−0.386).

**Table 3.** Correlations between serum and joint fluid study drug concentrations with changes of VAS and percentage of changes of cytokines and receptors from 1^st^ to 2^nd^ visit. Negative values correspond to negative correlation of the variable with the drug concentration.

**Variables correlated to serum and JF drug concentrations**	**Celecoxib group**				**Etoricoxib group**			
	**serum**		**JF**		**serum**		**JF**	
	**r**	**p**	**r**	**p**	**r**	**p**	**R**	**p**
**VAS**	0.551	0.172	−0.078	0.911	−0.016	0.929	0.334	0.184
**IL-1β S**	0.324	0.077	−0.174	0.539	0.247	0.866	0.209	0.358
**IL-1 β JF**	−0.085	0.166	0.238	**0.032**	−0.096	0.171	−0.386	**0.005**
**IL-1Ra S**	0.641	0.639	−0.163	0.857	0.027	0.881	−0.24	0.729
**IL-1Ra JF**	−0.151	0.425	0.451	0.071	−0.218	0.296	0.031	0.921
**IL-6 S**	−0.068	0.747	0.102	0.51	−0.355	0.202	−0.34	0.174
**IL-6 JF**	0.615	0.096	−0.09	0.952	0.104	0.228	−0.166	0.328
**TNF-α S**	0.355	0.112	−0.184	0.458	−0.247	0.133	−0.221	0.236
**TNF-α JF**	0.092	0.606	−0.153	0.192	−0.029	0.56	−0.222	0.439
**sTNFR1 S**	0.182	0.051	0.113	0.93	−0.248	0.343	−0.093	0.978
**sTNFR1 JF**	0.085	0.486	0.613	0.121	−0.193	0.501	0.411	0.103
**sTNFR2 S**	0.387	0.534	0.103	0.493	0.322	0.286	0.255	0.358
**sTNFR2 JF**	0.282	**0.028**	0.218	0.616	0.095	0.573	0.395	0.178

s: soluble, S: serum, JF: joint fluid, r: correlation coefficient

**Table 4** shows correlations of synovial fluid penetration index with cytokines and receptors and pain VAS changes between 1^st^ and 2^nd^ visit. Statistical significant relationship was noticed with synovial fluid IL-1β (p=0.03, correlation coefficient 0.39) for the celecoxib group, and with synovial fluid sTNFR1 receptor (p=0.02, correlation coefficient 0.053) for the etoricoxib group.

**Table 4.** Correlations of celecoxib and etoricoxib synovial fluid penetration index, with pain VAS and percentage of cytokine and receptors changes, between 1^st^ and 2^nd^ visit. Negative values correspond to negative correlation of the variable with the penetration index.

**Variables correlated to penetration index**	**Celecoxib group**		**Etoricoxib group**	
	**Correlation Coefficient r**	**p**	**Correlation Coefficient r**	**p**
**VAS**	−0.23	0.91	−0.01	0.65
**IL-1β S**	−0.22	0.58	0.1	0.42
**IL-1β JF**	0.39	**0.03**	−0.37	0.24
**IL-1Ra S**	−0.13	0.87	−0.27	0.47
**IL-1Ra JF**	0.44	0.1	0.17	0.66
**IL-6 S**	0.25	0.61	−0.1	0.35
**IL-6 JF**	−0.17	0.96	−0.22	0.78
**TNF-α S**	−0.24	0.39	−0.1	0.91
**TNF-α JF**	−0.21	0.19	−0.23	0.54
**sTNFR1 S**	0.03	0.85	0.1	0.6
**sTNFR1 JF**	0.43	0.17	0.53	**0.02**
**sTNFR2 S**	0.1	0.58	0.05	0.78
**sTNFR2 JF**	−0.02	0.74	0.3	0.37

s: soluble, S: serum, JF: joint fluid

## DISCUSSION

In the current study, we explored the effect of etoricoxib and celecoxib on the synovial fluid and serum concentrations of several cytokines in patients with inflammatory arthritis and knee joint effusion. To the best of our knowledge, this is the first study of this type investigating the impact of these drugs on synovial membrane penetration index of any NSAID with cytokine changes during treatment or with change in pain VAS in humans.

In our study, both celecoxib and etoricoxib caused the reduction of IL-1β levels in serum and SF, which was borderline statistically significant only in the etoricoxib group and only in serum. Moreover, celecoxib increased IL-1Ra SF levels. IL-1β levels in SF in the control group were increased as well. When comparing celecoxib with control group, we note a statistically significant reduction of IL-1β levels in serum and SF and increase of synovial fluid IL-1Ra in celecoxib group. Our findings concerning the reduction of IL-1β are in agreement with the only published in vivo study on the action of 200 mg daily Celecoxib on IL-6. Alvarez et al. published that the expression of IL-1β on synovial membrane was reduced significantly with celecoxib.^14^ Most of the published results regarding the effect of NSAIDs on IL-1β are in agreement with our results: indomethacin,^15^ thioprofenic acid, flurbiprofen^16^ and aspirin 300 mg daily^17^ reduced IL-1β, whereas aspirin 80 mg had no effect on it. Ghezzi et al. 1998 published that S-ketoprofen increases IL-1 production in animal studies.^18^ Published results on the action of NSAIDs on IL-1Ra are scarce: both ibuprofen^19^ and diclofenac^20^ lines increase the expression of IL-1Ra in cell lines, whereas aspirin at a small dose in vivo and in vitro^21^ has no action. There are no published studies on the action of coxibs on IL-1 receptors.

In our study, IL-6 levels reduced statistical significantly in the celecoxib treated group both in serum and SF and in the etoricoxib treated group only in synovial fluid. In the control group, there was no statistical significant difference on this cytokine. Bianchi et al. results are in agreement with the current study.^22^ There has been no study on etoricoxib action on IL-6. Generally there are several studies on the effect of NSAIDs on IL-6, and the results of most of them agree with the findings of the present study: Kang et al. 2001, studied the influence of several NSAIDs on IL-6 and found that they all decrease its levels.^23^ Bianchi et al.^22^ compared the influence of nimesulid and celecoxib on IL-6 levels in SF, and noted the inhibitory action of both. An in vitro study demonstrated that aspirin in low to medium doses results in increase of IL-6 levels, while in high doses results in reduction of IL-6. In the same study indomethacin causes dose-related increase of IL-6. In another vivo study, etodolac significantly reduced SF IL-6.^24^

We found that TNF-α levels are not statistical significantly reduced both in serum and SF in celecoxib and etoricoxib treated group, but this reduction is statistically significant only in SF when comparing with the control group. Our results are in accordance with Alvarez et al. study, which is the only one on the effect of celecoxib 200 mg/daily on TNF-α in synovial membrane.^14^ Our results are also in agreement with an in vivo study in patients with schizophrenia which failed to show a reduction in serum TNF-α.^25^ Celecoxib in low doses in vitro reduces both TNF-α levels and NF-κB activity in cell lines, whereas it has the opposite effect in high doses.^2^ The results of several studies regarding the actions of various NSAIDs on TNF-α levels are conflicting: in some studies nimesulide,^26^ indomethacin,^27^ naproxen^28^ and ibuprofen^29^ lowered TNF-α levels, while in others indomethacin,^30^ S-ketoprofen^18^ and etodolac^24^ increased it. With regard to the effect of celecoxib and etoricoxib on soluble receptors of TNF-α, there is no data with the exception of rofecoxib where no action on sTNFR1 was noted.^4^ Because their role is rather to bind TNF and to inactivate the biological activities of TNF-α and TNF-β, it is tempting to speculate that the increase of sTNFR2 levels in synovial fluid that we observed in our study in the etoricoxib group, may diminish activity of TNF-α contributing to the anti-inflammatory action of the drug. It is worth noting that the method we used for the measurement of the soluble receptors, detects the sum of them, namely both the unbound and bound to TNF, sTNFR. The method of measurement of TNF-α levels we used detects the sum of TNF-α, namely both the unbound and the bound to sTNFR, TNF.

We noticed a positive correlation of penetration index with the percentage of decrease of SF IL-1β levels in celecoxib group, and a positive correlation of penetration index with the percentage of increase of SF sTNFR1 in etoricoxib group. These correlations depict the increase of celecoxib and etoricoxib anti-inflammatory action, with the increase of their penetration index. We know that there is a poor correlation between plasma concentration of an NSAID and its efficacy,^31^ whereas the SF concentrations seem to have better correlation with clinical efficacy.^32,33^ Moreover clinical efficacy of an NSAID seems to extend beyond that expected from half-life and this is probably due to the protracted decrease of inflammatory mediators in the joint.^34,35^ Our results are in agreement with this concept.

In conclusion, in our study, celecoxib decreases IL-1β and IL-6 serum and SF levels, SF TNF-α levels, and increases IL-1Ra SF levels, whereas there is no statistically significant change on sTNFR1 and sTNFR2, either in serum or in SF. Etoricoxib decreases serum and SF IL-1β levels, IL-6 SF levels, and SF TNF-α levels, also increases SF sTNFR2 levels, and has no effect on IL-1Ra levels. Comparing the action of the two drugs on cytokine levels we noticed no difference on IL-1β, IL-6 and TNF-α levels, whereas celecoxib increases SF IL-1Ra levels, and etoricoxib increases SF sTNFR2 levels with statistical significant difference. There is also a dose-dependent action of celecoxib and etoricoxib on the decrease of SF IL-1β, but for etoricoxib there is a possible “ceiling effect” for this action. Moreover, celecoxib has a dose-dependent action on the increase of synovial fluid sTNFR2 levels. This is the first in vivo study in humans comparing celecoxib and etoricoxib action on pro-inflammatory cytokines and their soluble receptors in serum and synovial fluid.
